# Supplementary material for: Engineering a synthetic energy-efficient formaldehyde assimilation cycle in Escherichia coli
Source: Nat Commun. 2023 Dec 20;14:8490. doi: 10.1038/s41467-023-44247-2 (PMC10733421; doi:10.1038/s41467-023-44247-2)
Supplement: Supplementary file 1 — Supplementary Information [file 41467_2023_44247_MOESM1_ESM.pdf]

Engineering a synthetic energy-efficient formaldehyde assimilation cycle in *Escherichia coli*

Wu *et al.*

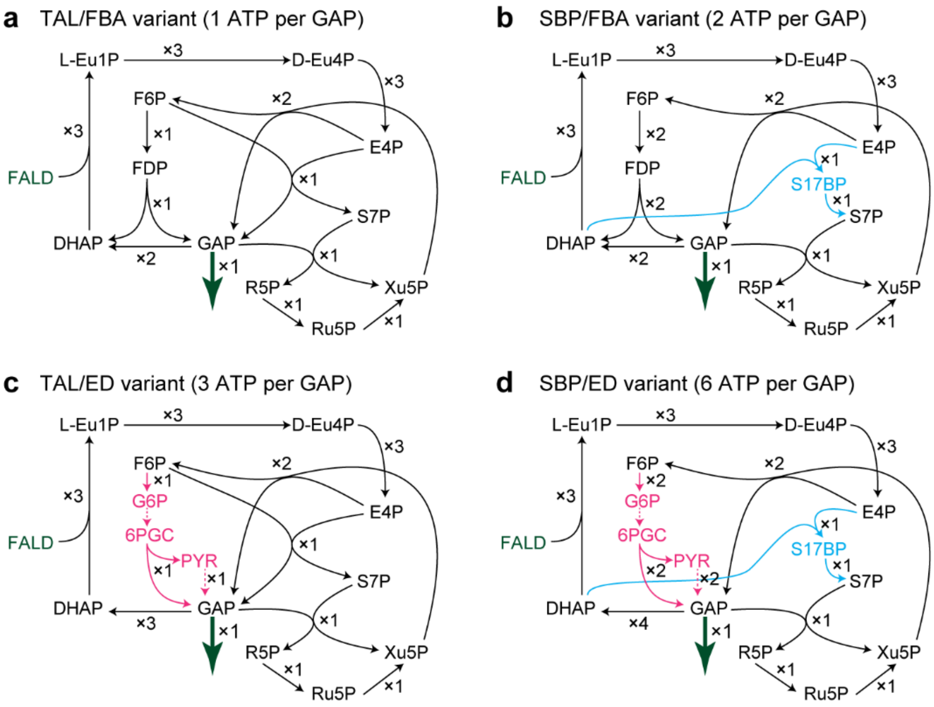

Supplementary Figure 1. Variants of the EuMP cycle. The EuMP cycle can be assembled from different metabolic routes: transaldolase (TAL) route or sedoheptulose 1,7-bisphosphatase (SBP) route for carbon rearrangement and fructose 1,6-bisphosphate aldolase (FBA) route or Entner-Doudoroff (ED) route for C6-sugar cleavage, resulting in four cycle variants (**a**) TAL/FBA, (**b**) SPB/FBA, (**c**) TAL/ED, and (**d**) SBP/ED variant. The TAL/FBA variant (**a**) has the least ATP cost, therefore it is the most energy efficient variant.

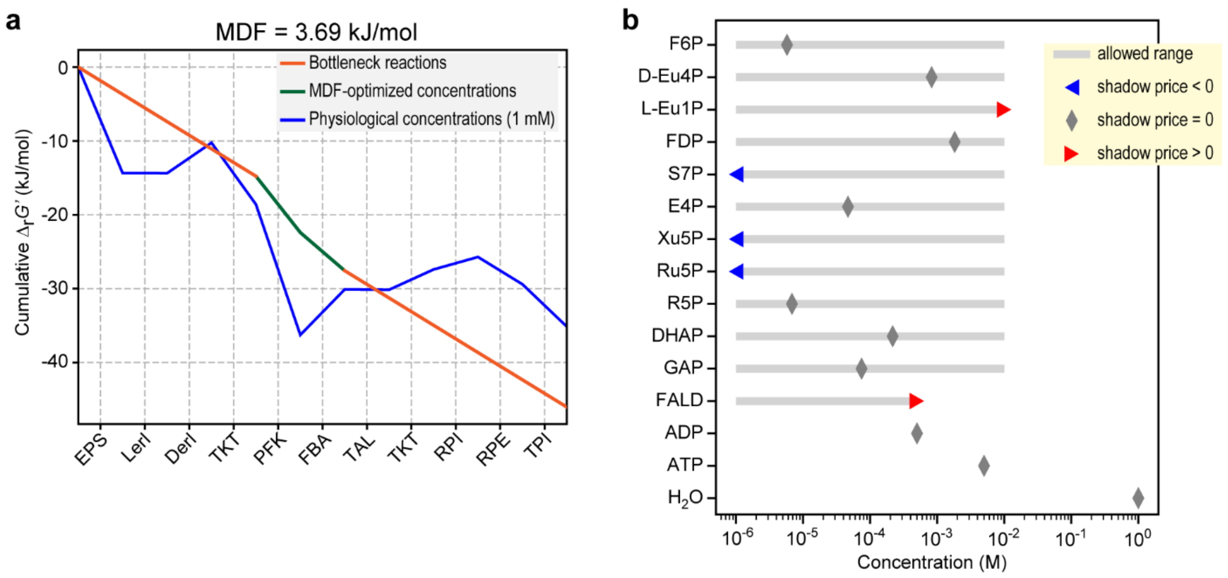

Supplementary Figure 2. Optimized thermodynamic profiles and Max-min Driving Force (MDF) analysis. (**a**) shows energetic profile of EuMP cycle. Blue lines correspond to $\Delta_r G'^m$ values of pathway reactions at pH 7.5. Green lines correspond to $\Delta_r G'$ values of pathway reactions after MDF optimization. The orange lines represent the predicted bottleneck reactions. (**b**) shows the metabolite concentrations, within physiological range (see Methods), after MDF optimization. Compound whose concentration limits the driving force, according to the MDF analysis, are shown in triangle.

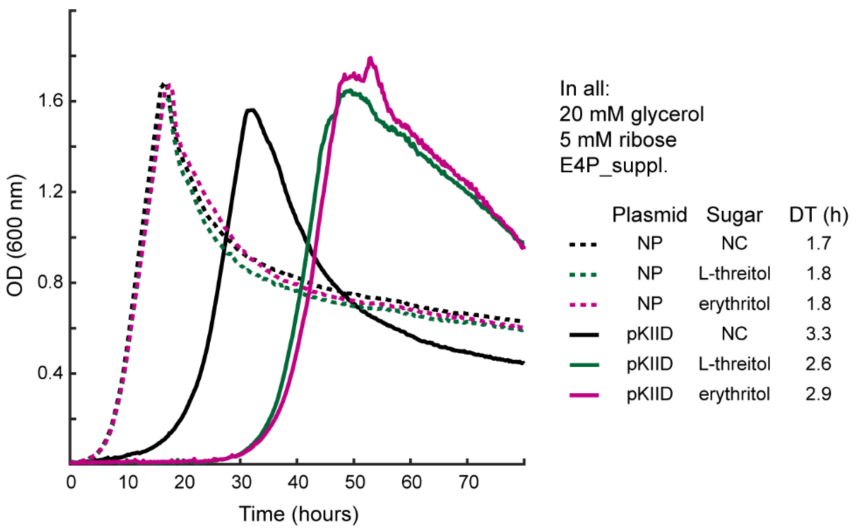

Supplementary Figure 3. Tetritol catabolism intermediates caused growth lag. Under relaxing conditions (complete media with E4P_suppl.), both 10 mM L-threitol and 10 mM erythritol were not toxic to *E. coli* (dashed lines). Plasmid pKIID maintenance and expression decreased growth rate (solid black line, no lag). Tetritol catabolism caused growth lag (solid green and pink lines). See the metabolic scheme in Fig. 2a. The plasmid pKIID has a constitutive promoter (Supplementary Table 1). Source data are provided as a Source Data file.

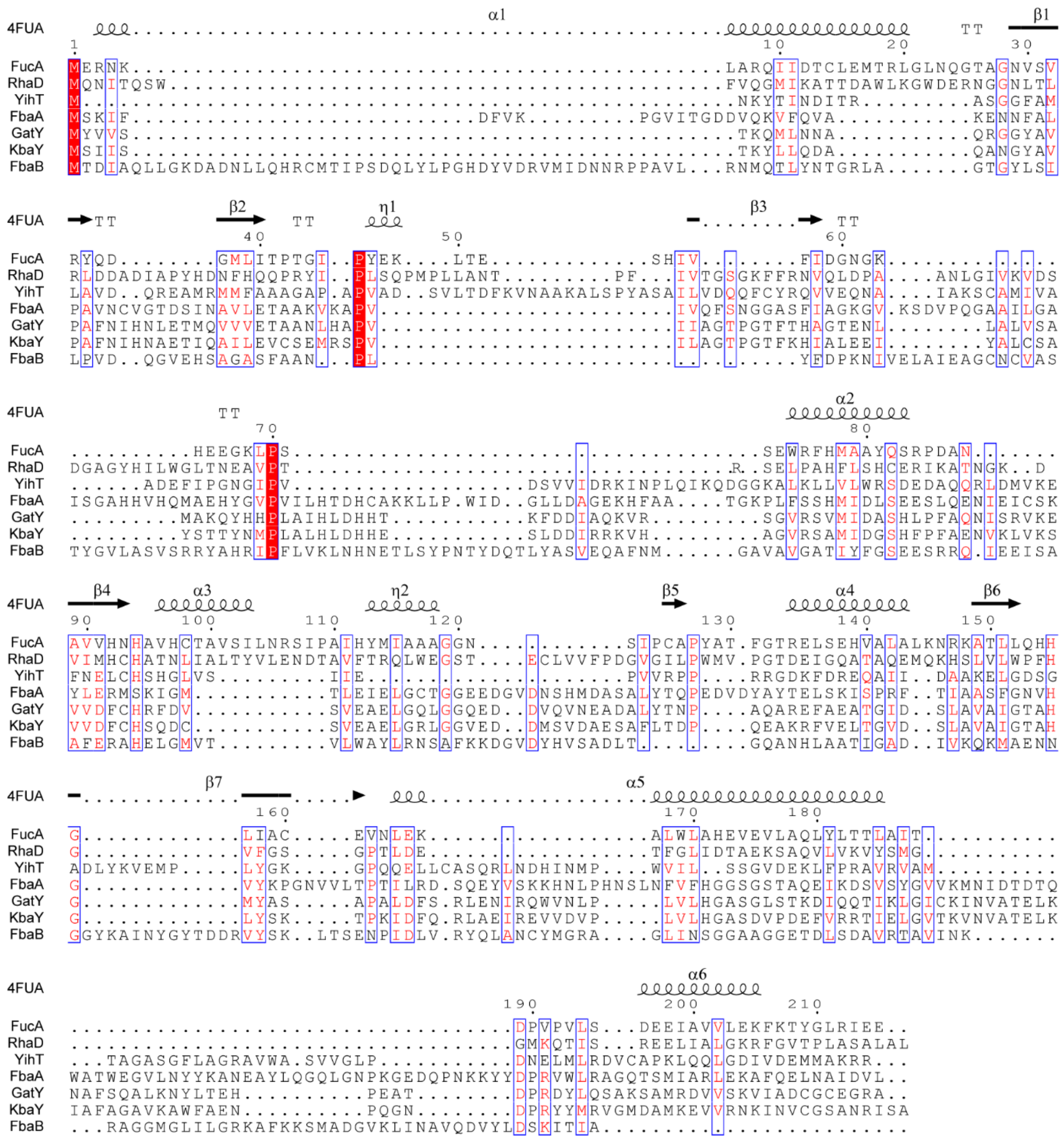

Supplementary Figure 4. Multiple sequence alignment of EPS candidates. Protein sequence of FucA (P0AB87), RhaD (P32169), YihT (P32141), FbaA (P0AB71), GatY (P0C8J6), KbaY (P0AB74) and FbaB (P0A991) were obtained from UniProt. Sequence alignment was produced by MAFFT[1]. ESPpript 3.0[2] was used for displaying the aligned sequences with the 3D structure of FucA, 4FUA[3]. Its α-helixes and β-sheets are indicated above.

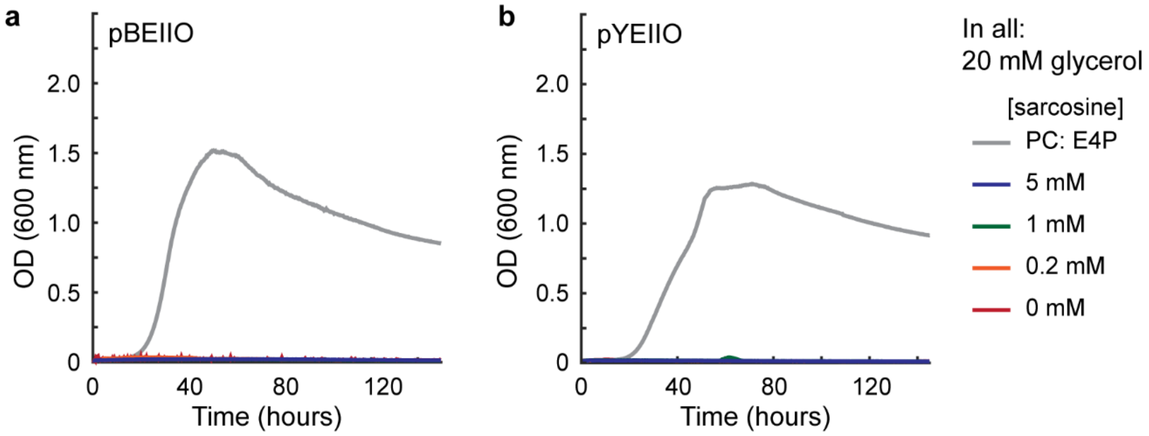

Supplementary Figure 5. FbaA and YihT failed enabling the growth from EuMP. The plasmids pBEIIO (**a**) and pYEIIO (**b**) (see Supplementary Table 1) cannot support growth of the ΔfrmTKT strain (see Supplementary Table 1) on sarcosine as E4P source. Source data are provided as a Source Data file.

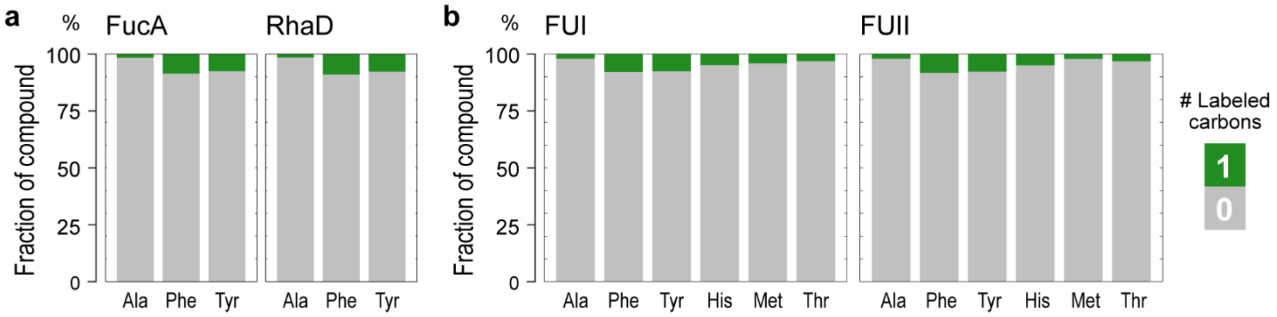

Supplementary Figure 6. Negative controls of the ^{13}C-labeling experiments. Panels **a** and **b** are control experiments under unlabeled sarcosine (ambient ^{13}C abundance) conditions for Fig. 3h and Fig. 4d, respectively. Source data are provided as a Source Data file.

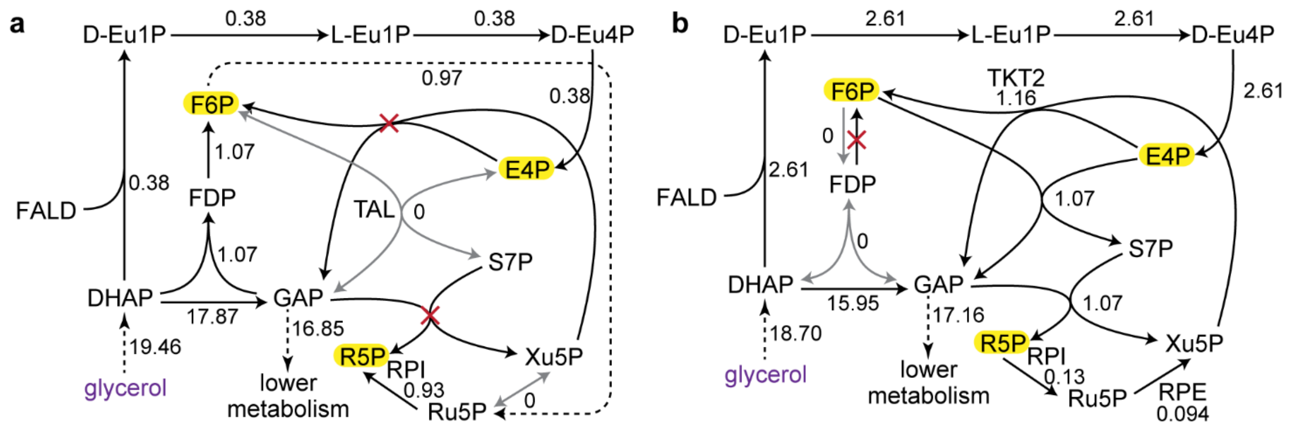

Supplementary Figure 7. Flux balance analysis showcases flux distributions of the EuMP within the ΔfrmTKT (a) and ΔFBP/GlpX (b) selections.

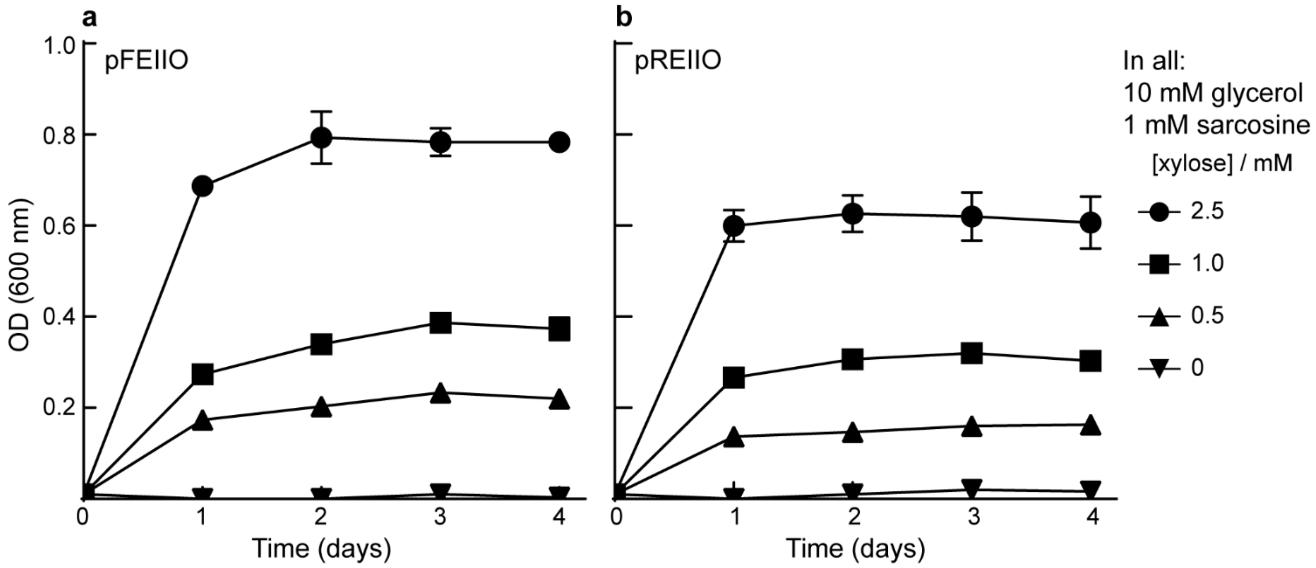

Supplementary Figure 8. Xylose is required to sustain the growth of the ΔFBP/GlpX pFEIIO (a) and pREIIO (b) strains. Error bar represents standard deviation, N = 3. Source data are provided as a Source Data file.

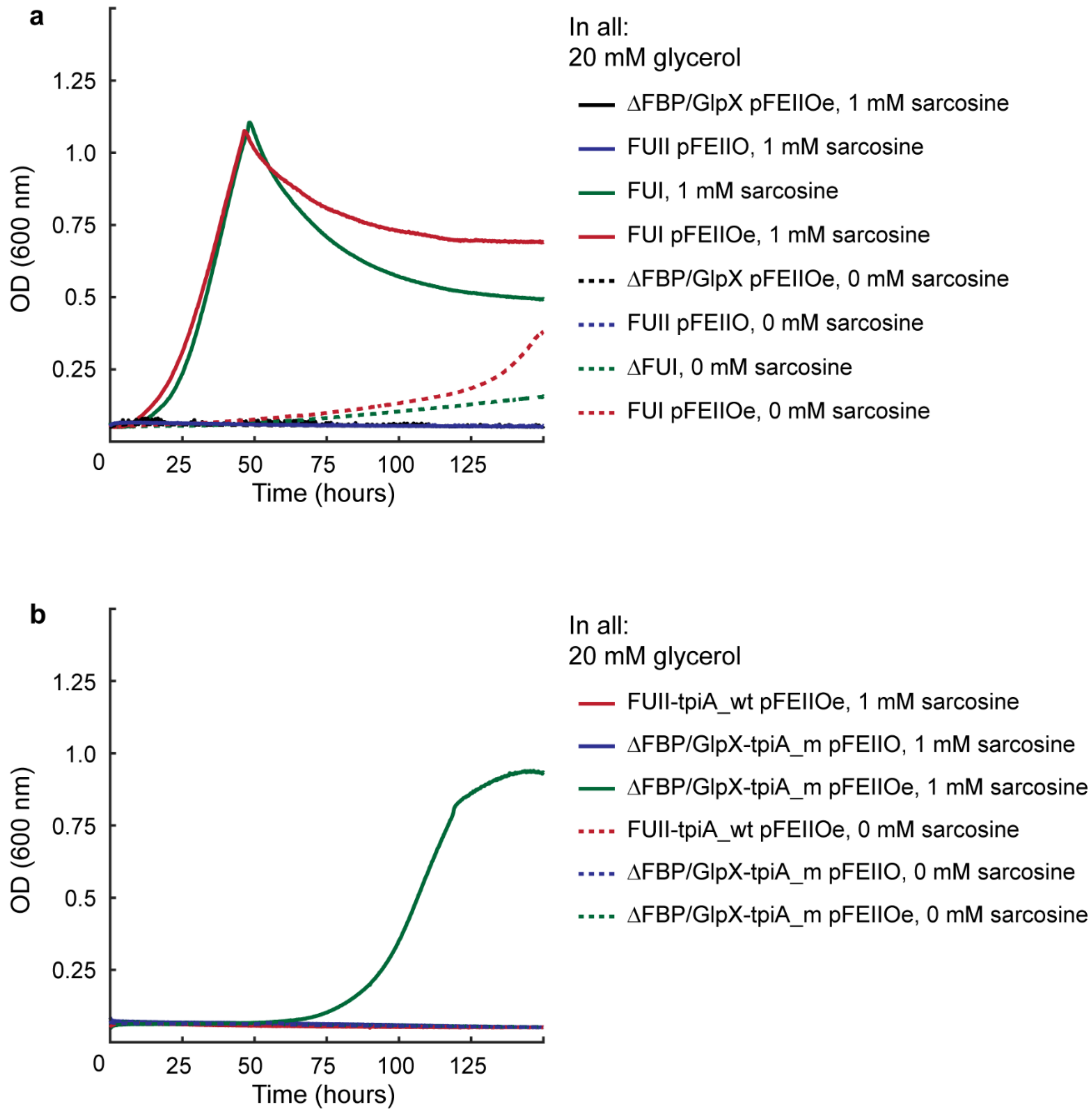

Supplementary Figure 9. Growth profiles of strains with reverse-engineered *lerI* (a) or *tpiA* (b) mutations. Strains and plasmids are listed in Supplementary Table 1. Source data are provided as a Source Data file.

Supplementary Table 1. List of strains and plasmids.

Strain	Genotype	Source
MG1655	K-12 $F^-\lambda^-$ *ilvG*$^-$*rfb*-50 *rph*-1	Lab collection
SIJ488	MG1655 Tn7::para-exo-beta-gam; prha-FLP; xylSpm-IsceI	[4]
DH5α	F^- *endA1 glnV44 thi-1 recA1 relA1 gyrA96 deoR nupG purB20* φ80dlacZΔM15 Δ(lacZYA-argF)U169, hsdR17($r_K^-m_K^+$), λ^-	Lab collection
ΔtktAB	SIJ488 *ΔtktA ΔtktB*	[5]
ΔfrmTKT	SIJ488 *ΔfrmRAB ΔtktA ΔtktB*	This study
ΔFBP/GlpX	SIJ488 *ΔfrmRAB Δfbp ΔglpX ΔmgsA*	This study
FUI1	Isolate of ΔFBP/GlpX pFEIIO after ALE	This study
FUI2	Isolate of ΔFBP/GlpX pFEIIO after ALE	This study
FUI3	Isolate of ΔFBP/GlpX pFEIIO after ALE	This study
FUII2	Isolate of ΔFBP/GlpX pFEIIO after ALE	This study
FUII3	Isolate of ΔFBP/GlpX pFEIIO after ALE	This study
ΔFBP/GlpX-tpiA_m	ΔFBP/GlpX *tpiA** (G94D)	This study
tpiA::Cm	ΔFBP/GlpX, Cm inserted downstream of *tpiA*	This study
FUII-tpiA_wt	FUII2, reverted *tpiA* to WT	This study
**Plasmids**	**Genotype**	**Source**
pZASS	p15A ori; $Strep^R$; P_{pgi-20}	Lab collection
pZASM	p15A ori; $Strep^R$; P_{pgi-10}	Lab collection
pKIID	pZASM:: *lerK, lerI, derI, eltD*	This study
pEIIO	pZASS:: *eryC, lerI, derI, soxA*	This study
pRIIO	pZASS:: *rhaD, lerI, derI, soxA*	This study
pREIIO	pZASS:: *rhaD, eryC, lerI, derI, soxA*	This study
pFEIIO	pZASS:: *fucA, eryC, lerI, derI, soxA*	This study
pBEIIO	pZASS:: *fbaA, eryC, lerI, derI, soxA*	This study
pYEIIO	pZASS:: *yihT, eryC, lerI, derI, soxA*	This study
pFEIIOe	pZASS:: *fucA, eryC, lerI* (I20L)*, derI, soxA*	This study
pKDsgRNA-tpiA	pKDsgRNA; $Strep^R$; tpiA guide RNA sequence	This study
pCas9cr4	p15A ori; Cm^R; P_{tet}-Cas9	[6]
pKDsgRNA-p15	pKDsgRNA; $Strep^R$; p15A guide RNA sequence	[6]

Supplementary references

1. Katoh, K. & Standley, D. M. MAFFT multiple sequence alignment software version 7: Improvements in performance and usability. *Mol Biol Evol* **30**, 772–80 (2013).
2. Robert, X. & Gouet, P. Deciphering key features in protein structures with the new ENDscript server. *Nucleic Acids Res* **42**, W320-4 (2014).
3. Dreyer, M. K. & Schulz, G. E. Catalytic mechanism of the metal-dependent fuculose aldolase from *Escherichia coli* as derived from the structure. *J. Mol. Biol.* **259**, 458–466 (1996).
4. Jensen, S. I., Lennen, R. M., Herrgard, M. J. & Nielsen, A. T. Seven gene deletions in seven days: Fast generation of *Escherichia coli* strains tolerant to acetate and osmotic stress. *Sci Rep* **5**, 17874 (2015).
5. Krusemann, J. L. *et al.* Artificial pathway emergence in central metabolism from three recursive phosphoketolase reactions. *FEBS J.* **285**, 4367–4377 (2018).
6. Reisch, C. R. & Prather, K. L. J. Scarless Cas9 assisted recombineering (no-SCAR) in *Escherichia coli*, an easy-to-use system for genome editing. *Curr Protoc Mol Biol* **117**, 31 8 1-31 8 20 (2017).
