# Supplementary material for: Engineering a synthetic energy-efficient formaldehyde assimilation cycle in Escherichia coli
Source: Nat Commun. 2023 Dec 20;14:8490. doi: 10.1038/s41467-023-44247-2 (PMC10733421; doi:10.1038/s41467-023-44247-2)
Supplement: Supplementary file 3 — Description of Additional Supplementary Files [file 41467_2023_44247_MOESM3_ESM.docx]

**Description of Additional Supplementary Files**

File Name: Supplementary Data 1

Description: Identified mutations of the parent and evolved ΔFBP/GlpX strains from NGS.

File Name: Supplementary Data 2

Description: Oligo primers used in the study.

File Name: Supplementary Data 3

Description: Sequences of heterologous expressed genes.
