# Supplementary material for: Engineering a synthetic energy-efficient formaldehyde assimilation cycle in Escherichia coli
Source: Nat Commun. 2023 Dec 20;14:8490. doi: 10.1038/s41467-023-44247-2 (PMC10733421; doi:10.1038/s41467-023-44247-2)
Supplement: Supplementary file 7 — Reporting Summary [file 41467_2023_44247_MOESM7_ESM.pdf]

nature portfolio

Corresponding author(s): Hai He

Last updated by author(s): Nov 26, 2023

Reporting Summary

Nature Portfolio wishes to improve the reproducibility of the work that we publish. This form provides structure for consistency and transparency in reporting. For further information on Nature Portfolio policies, see our Editorial Policies and the Editorial Policy Checklist.

Statistics

For all statistical analyses, confirm that the following items are present in the figure legend, table legend, main text, or Methods section.

n/a	Confirmed	
☐	☒	The exact sample size (*n*) for each experimental group/condition, given as a discrete number and unit of measurement
☐	☒	A statement on whether measurements were taken from distinct samples or whether the same sample was measured repeatedly
☒	☐	The statistical test(s) used AND whether they are one- or two-sided<br>*Only common tests should be described solely by name; describe more complex techniques in the Methods section.*
☒	☐	A description of all covariates tested
☒	☐	A description of any assumptions or corrections, such as tests of normality and adjustment for multiple comparisons
☐	☒	A full description of the statistical parameters including central tendency (e.g. means) or other basic estimates (e.g. regression coefficient) AND variation (e.g. standard deviation) or associated estimates of uncertainty (e.g. confidence intervals)
☒	☐	For null hypothesis testing, the test statistic (e.g. *F*, *t*, *r*) with confidence intervals, effect sizes, degrees of freedom and *P* value noted<br>*Give P values as exact values whenever suitable.*
☒	☐	For Bayesian analysis, information on the choice of priors and Markov chain Monte Carlo settings
☒	☐	For hierarchical and complex designs, identification of the appropriate level for tests and full reporting of outcomes
☒	☐	Estimates of effect sizes (e.g. Cohen's *d*, Pearson's *r*), indicating how they were calculated

Our web collection on statistics for biologists contains articles on many of the points above.

Software and code

Policy information about availability of computer code

Data collection	Growth data were measured by Gen5 v3; LC-MS data were collected and processed with Thermo Scientific Xcalibur v4.1.31.9
Data analysis	The source code of FBA and MDF analysis are deposited on Github (https://github.com/he-hai/PubSuppl). breseq (v0.31.0) was used for NGS data analysis. Further software used: MatLab, python v3.8.5, cobrapy v0.20.0, equilibrator-api v0.4.7 and equilibrator-pathway v0.4.7.

For manuscripts utilizing custom algorithms or software that are central to the research but not yet described in published literature, software must be made available to editors and reviewers. We strongly encourage code deposition in a community repository (e.g. GitHub). See the Nature Portfolio guidelines for submitting code & software for further information.

Data

Policy information about availability of data

All manuscripts must include a data availability statement. This statement should provide the following information, where applicable:

- Accession codes, unique identifiers, or web links for publicly available datasets
- A description of any restrictions on data availability
- For clinical datasets or third party data, please ensure that the statement adheres to our policy

Raw reads of NGS are deposited at NCBI and can be accessed under BioProject PRJNA895983 [https://www.ncbi.nlm.nih.gov/bioproject/PRJNA895983/]. MG1655 genome is from Genbank: NC_000913 [https://www.ncbi.nlm.nih.gov/nuccore/NC_000913]. Protein sequences of DHAP aldolases from E. coli are obtained from UniProt, FucA P0AB87 [https://www.uniprot.org/uniprotkb/P0AB87], RhaD P32169 [https://www.uniprot.org/uniprotkb/P32169], YihT P32141 [https://

www.uniprot.org/uniprotkb/P32141], FbaA P0AB71 [https://www.uniprot.org/uniprotkb/P0AB71], GatY P0C8J6 [https://www.uniprot.org/uniprotkb/P0C8J6], KbaY P0AB74 [https://www.uniprot.org/uniprotkb/P0AB74] and FbaB P0A991 [https://www.uniprot.org/uniprotkb/P0A991]. Source data are provided with this paper.

Research involving human participants, their data, or biological material

Policy information about studies with human participants or human data. See also policy information about sex, gender (identity/presentation), and sexual orientation and race, ethnicity and racism.

Reporting on sex and gender	N/A
Reporting on race, ethnicity, or other socially relevant groupings	N/A
Population characteristics	N/A
Recruitment	N/A
Ethics oversight	N/A

Note that full information on the approval of the study protocol must also be provided in the manuscript.

Field-specific reporting

Please select the one below that is the best fit for your research. If you are not sure, read the appropriate sections before making your selection.

☒ Life sciences ☐ Behavioural & social sciences ☐ Ecological, evolutionary & environmental sciences

For a reference copy of the document with all sections, see nature.com/documents/nr-reporting-summary-flat.pdf

Life sciences study design

All studies must disclose on these points even when the disclosure is negative.

Sample size	No sample size determination was performed. Growth curves used duplicates, triplicates, or quadruplicates. Shown results are single experiments representative of repetitions showing similar results. 13C-labeling used triplicates or quadruplicates. ALE culture were two independent culture.
Data exclusions	Data was excluded when clear outliers were observed. Criteria for data exclusion where not pre-established.
Replication	Reproducibility was verified by conducting the independent experiments at least two times with freshly prepared cells from cryo stock.
Randomization	Since this study was conducted using single microorganism in the controlled laboratory environment and focused on its performance, the allocation is not relevant to this study.
Blinding	The blinding was not possible for us since all measurement was performed either by plate reader, which reads OD automatically, or by LC-MS. The blinding would not be applicable.

Reporting for specific materials, systems and methods

We require information from authors about some types of materials, experimental systems and methods used in many studies. Here, indicate whether each material, system or method listed is relevant to your study. If you are not sure if a list item applies to your research, read the appropriate section before selecting a response.

Materials & experimental systems

n/a	Involved in the study	
☒	☐	Antibodies
☒	☐	Eukaryotic cell lines
☒	☐	Palaeontology and archaeology
☒	☐	Animals and other organisms
☒	☐	Clinical data
☒	☐	Dual use research of concern
☒	☐	Plants

Methods

n/a	Involved in the study	
☒	☐	ChIP-seq
☒	☐	Flow cytometry
☒	☐	MRI-based neuroimaging

Plants

Seed stocks	N/A
Novel plant genotypes	N/A
Authentication	N/A
